# Combinations of immuno-checkpoint inhibitors predictive biomarkers only marginally improve their individual accuracy

**DOI:** 10.1186/s12967-019-1865-8

**Published:** 2019-04-23

**Authors:** Matteo Pallocca, Davide Angeli, Fabio Palombo, Francesca Sperati, Michele Milella, Frauke Goeman, Francesca De Nicola, Maurizio Fanciulli, Paola Nisticò, Concetta Quintarelli, Gennaro Ciliberto

**Affiliations:** 1 SAFU Unit, IRCCS Regina Elena National Cancer Institute, Rome, Italy; 2 Unit of Biostatistics and Clinical Trials, Istituto Scientifico Romagnolo per lo Studio e la Cura dei Tumori (IRST) IRCCS, Meldola, Italy; 3 Takis srl, Rome, Italy; 4 UOS Biostatistics, IRCCS Regina Elena National Cancer Institute, Rome, Italy; 5 Medical Oncology 1, IRCCS Regina Elena National Cancer Institute, Rome, Italy; 6 UOSD Oncogenomics and Epigenetics, IRCCS Regina Elena National Cancer Institute, Rome, Italy; 7 UOSD Immunology and Immunotherapy Unit, IRCCS Regina Elena National Cancer Institute, Rome, Italy; 8 Department of Paediatric Haematology, IRCCS Ospedale Pediatrico Bambino Gesù, Rome, Italy; 9 IRCCS Regina Elena National Cancer Institute, Rome, Italy

**Keywords:** Immuno-checkpoint inhibitors biomarkers, Genomics, Immunotherapy, ImmunoPhenoScore, TIDE, RNA-seq, Exome sequencing, Majority voting, Generalized linear models

## Abstract

**Background:**

There are no accepted universal biomarkers capable to accurately predict response to immuno-checkpoint inhibitors (ICI). Although recent literature has been flooded with studies on ICI predictive biomarkers, available data show that currently approved companion diagnostics either leave out many possible responders, as in the case of PD-L1 testing for first-line metastatic lung cancer, or apply to a small subset of patients, such as the recently approved treatment for microsatellite instability-high or mismatch repair deficiency tumors. In this study, we conducted a survey of the available data on ICI trials with matched genomic or transcriptomic datasets in order to cross-validate the proposed biomarkers, to assess whether their prediction power was confirmed and, mainly, to investigate if their combination was able to generate a better predictive tool.

**Methods:**

We extracted clinical information and sequencing data details from publicly available datasets, along with a list of possible biomarkers obtained from the recent literature. After an operation of data harmonization, we validated the performance of all the biomarkers taken individually. Furthermore, we tested two strategies to combine the best performing biomarkers in order to improve their predictive value.

**Results:**

When considered individually, some of the biomarkers, such as the ImmunoPhenoScore, and the IFN-γ signature, did not confirm their originally proposed predictive power. The best absolute scoring biomarkers are TIDE, one of the ICB resistance signatures and CTLA4 with a mean AUC > 0.66. Among the combinations tested, generalized linear models showed the best performance with an AUC of 0.78.

**Conclusions:**

We confirmed that the available biomarkers, taken individually, fail to provide a satisfactory predictive value. Unfortunately, also combination of some of them only provides marginal improvements. Hence, in order to generate a more robust way to predict ICI efficacy it is necessary to analyze and combine additional biomarkers and interrogate a wider set of clinical data.

**Electronic supplementary material:**

The online version of this article (10.1186/s12967-019-1865-8) contains supplementary material, which is available to authorized users.

## Background

Tumors escape from immunological control by activating during their growth and development a wealth of negative immune-checkpoints of which the best characterized and clinically validated are B7/CTLA4 and PD-L1/PD-L2/PD1 [1]. These checkpoints prevent the priming and/or activation of tumor-infiltrating lymphocytes, activate regulatory T cells or cause T cell exhaustion, which all together facilitate unchecked tumor growth. Monoclonal antibodies (mAb) such as anti-CTLA4, anti-PD-1 and anti-PD-L1 were developed to specifically target these pathways. Over the last years, clinical development of these antibodies have led to unprecedented outcomes characterized by increased overall survival and long term disease control for metastatic cancer patients in a growing number of indications [2, 3]. Currently, the US FDA and EU have approved the commercialization of these immuno-checkpoint inhibitors (ICIs) in several metastatic solid tumors. Also one of those inhibitors was recently approved for any metastatic solid tumor with microsatellite instability-high (MSI-H) or mismatch repair deficiency (dMMR), the first drug approval based on genetic features shared across different tumor types [4].

Despite these major breakthroughs, responses to immunotherapy are observed only in a minority of patients, and some biomarkers approved as companion diagnostics to stratify patients only marginally help to select responders from non-responders. As an example, patients are currently eligible for treatment with anti-PD1 mAb pembrolizumab as first-line treatment of metastatic non-small-cell lung cancer (mNSCLC) if their tumor cells result ≥ 50% positive to immunohistochemistry (IHC) analysis of ligand PD-L1 expression. As a matter of fact, according to a recent meta-analysis, the percentage of responders in PD-L1-negative patients is not strongly dissimilar from that of the PD-L1 positive group (23–29% vs 5–12%) [5]; this further supports the need for additional and more robust predictive biomarkers, and to integrate a more extensive amount of information into a final prediction score.

Along this line, the *ImmunoPhenoScore (IPS)*, an approach based on RNA-seq, was recently described as a novel system for the prediction of responders to ICI treatment in melanoma patients [6]. Relative expression levels of a number of intrinsic/extrinsic factors were combined (by addition or subtraction) in an algorithm computing a final score called the IPS. The whole model was built with the purpose of predicting the expression of Granzyme and Perforin genes as an indirect measure of immunogenicity. It was developed using TCGA data and validated by the sole available datasets in which ICI clinical data and transcriptomic profile were available, i.e. two melanoma datasets associated to anti-PD-1 and anti-CTLA4 treatment [7, 8]. The discriminating power of the IPS system looked very accurate (mostly for the anti-PD-1 dataset), where the overall responder group appeared to be clearly separated by a particular IPS cutoff (> 6).

Apart from this case, several discrete DNA/RNA-based biomarkers have emerged in recent literature. For instance, the study of inflammatory processes, which led to the identification of an interferon gamma (IFN-γ) signature, called T cell-inflamed GEP (Gene Expression Profile) [9]. The IFN-γ signature was more recently employed in a pan-cancer analysis of 300 patients treated with ICI, resulting in higher response rates when integrated with Tumor Mutational Burden (TMB), even if the authors did not report an AUC performance for this new combinatorial biomarker classifier [10]. Furthermore, animal modeling of the genetic status of *MLH1* and *POLE* genes confirmed the importance of the impairment of DNA mismatch repair (MMR) machinery [11], as expected. This was then able to increase the neoantigen load and sensitivity to ICI treatment [11]. Recently, the TIDE signature was introduced as a mixed T cell classification, partitioning patients into cytotoxic T lymphocyte (CTL) high (dysfunction score) or CTL low (exclusion score) [12]. Finally, it has been noted that epithelial–mesenchymal transition expression traits could represent the link between tumor intrinsic and tumor extrinsic factors, which lead to immune checkpoint blockade (ICB) resistance, resulting in the emergence of several possible biologic signatures [7, 13–20]. Starting from this background information, we conducted an in silico validation and combination analysis of the proposed biomarkers using publicly available datasets from ICI clinical trials and associated molecular annotations.

## Results

We collected genomic and/or transcriptomic data from anti-PD-1 and anti-CTLA4 studies with clinical information (Table 1). There is a clear imbalance regarding the tumor type distribution, with a strong prevalence of melanoma datasets (4) and only one dataset for NSCLC. Furthermore, the dimensionality of these datasets is quite small (mean of 39 and 66 patients for RNA and WES data, respectively), therefore making it difficult to infer a robust model.

**Table 1:** List of available studies

First author	Tumor type	RNA-seq data	WES mutation list	References
Hugo	Melanoma	28	26	[7]
Riaz	Melanoma	49	65	[20]
Rizvi	NSCLC	NA	33	[21]
Snyder	Melanoma	NA	63	[22]
Van Allen	Melanoma	41	105	[8]

We did not re-evaluate the expression levels via sequence re-analysis in order to create a whole comprehensive dataset, since the dramatic clinical confounding factors would have vanished our efforts (Additional file 1: Methods, Additional file 2: Table S1, Additional file 3: Table S2 and Additional file 4: Table S3).

A list of putative immunotherapy biomarkers computable from genomic and transcriptomic data (Table 2) was derived from the aforementioned datasets and other in silico data. For protein-based ones, we included their own RNA derivation, such as PD-L1 gene expression (CD274). These were included with the assumption that there was a strong correlation between the protein and its own RNA transcript levels, while being aware that there are other processes involved in the regulation of final protein expression levels (e.g. degradation, stabilization). On the other hand, our in-house RNA-sequencing of lung specimens demonstrates a strong correlation between the PD-L1 gene *FPKM* and the IHC protein level (CLONE 22C3, DAKO) (data not shown). We also included multi-gene markers such as the IFN-γ signature (originally computed via NanoString technology). All the genes included in the signature gene lists are listed in Table 3, with the exclusion of the algorithmic ones for which we refer to the original sources (e.g. TIDE and IPS). Furthermore, a high-level summary of the overall analysis process is provided (Additional file 5: Figure S1).

**Table 2:** List of biomarkers in this study

Marker	Origin	Type	Description	References
CD274	RNA	Single gene	Programmed death 1 ligand 1	[21]
Mutational load	DNA	Nr. mutations	Absolute number of mutations in WES	[8, 22]
IFN-y (reduced set)	RNA	Gene set	Interferon gamma reduced set	[9]
IFN-y (expanded set)	RNA	Gene set	Interferon gamma reduced set	[9]
IPS	RNA	Algorithm	Immunophenoscore	[6]
PDCD1	RNA	Single gene	Programmed death 1	[23]
POLE	RNA	Single gene	DNA polymerase epsilon, catalytic subunit	[11]
POLE2	RNA	Single gene	DNA polymerase epsilon 2, accessory subunit	[11]
POLE3	RNA	Single gene	DNA polymerase epsilon 3, accessory subunit	[11]
POLE4	RNA	Single gene	DNA polymerase epsilon 4, accessory subunit	[11]
CTLA4	RNA	Single gene	Cytotoxic T-lymphocyte associated protein 4	[25]
PDCD1LG2	RNA	Single gene	Programmed cell death 1 ligand 2	[24]
ICB resist. signature 1	RNA	Gene set	Epithelial–mesenchymal transition signature 1	[7]
ICB resist. signature 2	RNA	Gene set	Epithelial–mesenchymal transition signature 2	[17]
ICB resist. signature 3	RNA	Gene set	Epithelial–mesenchymal transition signature 3	[18]
AXL pathway	RNA	Gene set	AXL receptor tyrosine kinase pathway	[19]
AXL	RNA	Single gene	AXL receptor tyrosine kinase	[19]
TIDE	RNA	Algorithm	Tumor immune dysfunction and exclusion	[12]

**Table 3:** Gene lists for gene set signatures

*IFN-y (reduced set)*
CXCL10, CXCL9, HLA-DRA, IDO1, IFNG, STAT1
*IFN-y (expanded set)*
CCL5, CD2, CD3D, CD3E, CIITA, CXCL10, CXCL13, CXCR6, GZMB, GZMK, HLA-DRA, HLA-E, IDO1, IL2RG, LAG3, NKG7, STAT1, TAGAP
*ImmunoPhenoScore (IPS)*
ACAP1, ADRM1, AHSA1, AIM2, APOL3, ARHGAP10, ATM, ATP10D, B2 M, BIRC3, BRIP1, C1GALT1C1, C3AR1, CASP3, CASQ1, CCL20, CCL3L1, CCL4, CCL5, CCNB1, CCR2, CCR5, CCR7, CCT6B, CD14, CD160, CD2, CD27, CD274, CD300E, CD37, CD3D, CD3E, CD3G, CD55, CD69, CD72, CD86, CD8A, CETN3, CFLAR, CLEC5A, CMKLR1, CSE1L, CTLA4, CXCR4, DAPP1, DARS, DOCK9, DUSP2, ESCO2, ETS1, EXO1, EXOC6, EXOSC9, EZH2, FCGR2A, FCGR2B, FCGR3A, FCRL6, FERMT3, FLT3LG, FOXP3, GDE1, GEMIN6, GNLY, GPSM3, GPT2, GZMA, GZMH, GZMK, GZMM, HAPLN3, HAVCR2, HLA-A, HLA-B, HLA-C, HLA-DMB, HLA-DPA1, HLA-DPA1, HLA-DPB1, HLA-DPB1, HLA-E, HLA-F, IARS, ICOS, IDO1, IFI16, IL18BP, IL2RB, IL34, IL4R, ITGA4, ITGAL, ITGAM, KIF11, KNTC1, L1CAM, LAG3, LCK, LIME1, LIPA, LRP1, LRRC42, LTK, MARCO, MMP12, MNDA, MPZL1, MRC1, MS4A6A, NCOA4, NEFL, NFKBIA, NKG7, NUF2, PARVG, PDCD1, PDCD1LG2, PDGFRL, PELO, PIK3IP1, PLEK, PRC1, PRSS23, PSAP, PSAT1, PTGER2, PTGES2, PTGIR, PTGS1, PTRH2, REPS1, RGS1, RTKN2, S100A8, S100A9, SAMSN1, SCG2, SDPR, SELL, SETD7, SIGLEC14, SIGLEC6, SIK1, ST8SIA4, STAB 1, TAL1, TAP1, TAP2, TFEC, TIGIT, TIMM13, TIPIN, TPK1, TRAT1, TRIB2, UQCRB, USP9Y, WIPF1, ZAP70, ZCRB1
*ICB resistance signature 1*
ADAMTS7, AXL, COL12A1, COL8A1, FAP, FBLN1, INHBA, LOXL2, MMP1, MMP13, ROR2, TAGLN, TWIST2, WNT5A
*ICB resistance signature 2*
CNN1, COL3A1, MXRA7, SERPINF2
*ICB resistance signature 3*
CST2, LAMA3
*AXL pathway*
AXL, FLT1, FLT4, GAS6, KDR, MERTK, MET, RET, TEK, TYRO3

Taking into consideration the lack of uniqueness in the separation of patients in responder and non-responder groups (all the five studies from which we extrapolated data used different methods and response evaluation criteria, Additional file 1: Methods), we selected a method that also takes into consideration the overall survival of progressive disease patients. Moreover, it allowed a good balancing with regards to the number of samples between the two groups (ranging from 38% of responders with respect to the total number of patients, to 70%).

**Fig. 1:**
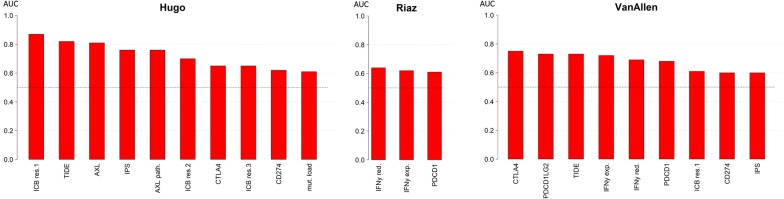
Overview of top scoring AUCs generated for all markers for the available studies with both DNA and RNA data. We plotted only tests with minimum AUC of 0.60, among 54 analyses (18 for each study), for readability purposes

We then computed the classification performance of all the proposed biomarkers in each available study. This resulted in 56 tests since there was a lack of RNA information in some datasets (Additional file 6: Table S4 and Fig. 1). The area under the curve (AUC) values markedly vary from 0.43 to 0.87. Of note, we could not reproduce the classification power of the IPS neither in the Hugo et al. [7] or in the Van Allen et al. [8] melanoma datasets (AUC 0.68 and 0.56 vs 1.00/1.00, using their same responder/non-responder separation types, respectively, as confirmed by [12]). TIDE signature performance was validated in the Hugo dataset (AUC 0.82) and slightly different in the Van Allen dataset (AUC 0.73 vs 0.80). This is due to their multi-class ROC with a third class on long-term survival patients, which we included in the responder group. However, we confirmed the published results with a three-class model (AUC 0.80). Finally, the performance of the IFN-γ reduced marker was slightly lower in our three transcriptomic datasets (AUC: 0.50, 0.64, 0.69) when compared to those of the published study (AUC 0.80, 0.66). This difference may reflect one of the technological biases of comparing RNA-seq results with Nanostring counts.

Moreover, we computed the mean AUC for all datasets for every classifier (Additional file 7: Table S5) to provide a better representation of every performance value among all studies. Therefore, every marker proposed in literature shows a discrete performance delta (0.01–0.35) when tested among different datasets, and, in general, the best performing parameters are TIDE, ICB resistance signature 1 and CTLA4 with a mean AUC of 0.71, 0.67 and 0.66, respectively. Observing the peak performances, TIDE, ICB signature 1 and AXL are the only individual markers with AUC > 0.8.

As a next step, we merged all the three datasets with transcriptomic data and the dichotomous marker predictions in a single whole dataset. This is necessary in order to have a dataset with a sufficient number of observations, which in turn increases statistical significance.

**Fig. 2:**
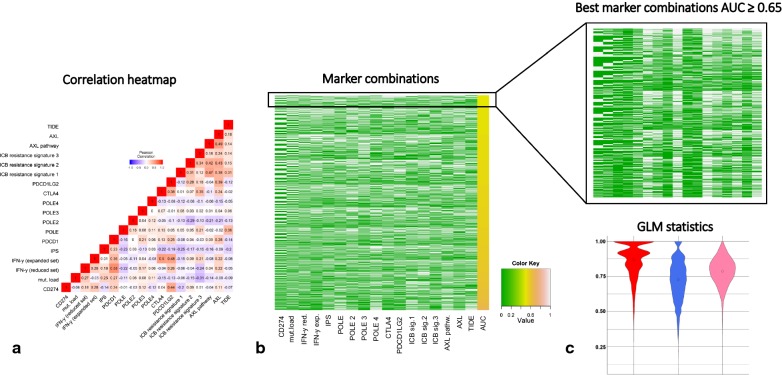
**a** Pearson correlation of all biomarkers in the RNA-seq studies; **b** heatmap representing the performance (yellow scale) of 4083 majority voting combinations with uncorrelated markers; **c** Violin plot of generalized linear models’ performance

Next, we tried to address the problem of correlation arising from markers that were linked or were function of others (e.g. PD-L1 and IPS, IFN-γ reduced and expanded sets). A Pearson correlation analysis (Additional file 8: Table S6 and Fig. 2a) showed that PD-1 gene and the IFN-γ reduced set are the only correlated markers at > 0.5 (at a 0.01 significance level). Notably, several parameters do not correlate significantly with the most accepted markers (e.g. IPS vs PD-L1, even if PD-L1 is one of the inputs of the IPS model), potentially suggesting that they could contain different information, and may benefit from a combinatorial approach.

In order to validate this hypothesis, we performed a majority voting exploration analysis in which we included all the uncorrelated markers, with the exclusion of PD-1 gene (Additional file 9: Table S7 and Fig. 2b).

All of these biomarker subsets were explored trying all possible combinations to find the best consensus group in 17, with 131,054 possibilities, according to the basic combinatory formula:

$$c = \mathop \sum \limits_{j = 2}^{n} \left( {\begin{array}{*{20}c} n \\ j \\ \end{array} } \right)$$

where *c* is the number of combinations and *n* is the number of markers.

The acceptable combinations in terms of AUC, defined as the percentage of those being equal to or better than 0.65 was then explored. For such analyses, we observed that the percentage of acceptable combinations is 2.1%. The frequencies of these uncorrelated markers in the acceptable combinations was computed (Additional file 10: Table S8): POLE4, ICB resistance signature 1 and TIDE are the most present, with frequency values of 0.68, 0.65 and 0.61, respectively.

Generalized linear models (GLMs) were then built as an additional strategy to combine these classifiers. We iterated cross-validation for 10,000-times, randomly selecting the 20% of the original set for testing. From the GLM predictions, we obtained mean AUC of 0.78, mean sensitivity of 0.87, mean specificity of 0.73 (Fig. 2c). Considering the significance of each coefficient, at 1% we had TIDE and ICB resistance signature 1, at 5% CTLA4 (Additional file 11: Table S9). It is noteworthy that TIDE and ICB resistance signature 1 have also been found among the top three most present markers of the best majority voting analyses and those with the best mean AUC when taken individually.

Taken together, these results confirm that the combination of multiple parameters enables us to improve the prediction power to a mean AUC of 0.71–0.78.

## Discussion

We explored whether several proposed RNA/DNA predictive biomarkers of efficacy of cancer immunotherapy with antibodies inhibitors of immunological checkpoints were confirmed by a cross-comparison among publicly available datasets. From these analyses we demonstrated that their combination led only to a modest improvement of the overall predictive performance. What we could learn from this study is the dramatic difficulty of putting the concept of immunotherapy response in a mathematical and statistical frame. The confounding factors here are hidden in several steps, namely the clinical classification, the patient history and the tumor sampling. The clinical classification, deriving from several different criteria, has already been discussed (see Additional file 1: Methods). The patient’s history is a determinant factor, since these molecular profiles are usually derived from tumor samples that have been collected months, if not years, before ICI therapy initiation. One can easily assume that during this often long period of time, also as a consequence of sequential non-ICI therapies (chemotherapy and target therapy), patients’ tumors have undergone clonal selection processes that have reshaped their molecular landscape. In a scenario of a biopsy with a time frame closer to the therapy, the tumor sampling effect comes at play. These issues have challenged researchers worldwide who are currently designing liquid biopsies strategies in order to find DNA/RNA biomarkers in the patient’s blood. This strategy would incredibly speed up the protocol if such biomarkers do exist and are actually detectable with novel highly sensitive technologies. Finally, a limitation of the currently employed –omic technologies is the lack of information about the spatial organization of the immune infiltrate, critical for an effective action of immune cells on the tumor mass. With the release of novel, larger and better annotated datasets one could stratify the patient population using all these criteria.

## Conclusions

We performed a validation study of a large panel of proposed biomarkers related to ICI response, in several solid tumors. Furthermore, a combination effort was carried out, leading to a slight increase in accuracy. We plan to carry on this study by expanding the patient cohort as additional data will become available, and to validate the top scoring biomarkers in selected tumor specimens.

## Methods

### Data collection

Clinical information and sequencing data from publicly available datasets was extracted. We searched GEO and PubMed records for genomic datasets matching the keyword “Immuno-Checkpoint Blockade”, “PD1”, “CTLA4”, “RNA-seq”, “Exome Sequencing”. We harmonized data in a list of patients with the relative response and for each patient we got the list of mutations derived by WES analysis and/or the list of expressed genes in TPM. All the scripts are written in R and are available at https://gitlab.com/bioinfo-ire-release/ici-biomarker-review, along with input datasets to reproduce all the singular and combinatorial analyses.

### Responder and non-responder separation

One of the main challenges of this work was to tackle the variability of how clinical benefit was measured in the different studies (Additional file 1: Methods). All the results shown in this paper are derived from the separation in which LB, CR, PR SD and PD with overall survival (when available) greater than 2 years are in the responder group, while NB and PD with overall survival lower than 2 years (or without this kind information) are in the non-responder group.

### Performance of individual markers

We used a partitioning system with the aim of building a receiver operating characteristics (ROC) curve for each biomarker in each study and set the threshold for each marker as the value for which the sum of sensitivity and specificity is maximized, according to Youden’s index. Basic statistics were then extracted: for each biomarker we joined the AUCs coming from different studies to have a minimum, a maximum and a mean value.

### Performance of combined markers

Before the combination step, we computed Pearson correlation coefficient among all the biomarkers. Then we excluded the minimum number of them in order to avoid correlation coefficients greater than 0.5. In the majority voting approach, we combined all the patients with all the uncorrelated biomarkers according to all the possible combinations starting from picking up just two of them to selecting all of them. We predicted a patient as responder whether at least half of the biomarkers agreed on that class. For each marker combination, we calculated confusion matrix, sensitivity, specificity and AUC, then we filtered out those with AUC < 0.65 and computed the frequency of each marker in these best combinations. Regarding GLM, we randomly selected a training set containing the 80% of the original set and built GLM models using the binomial family function (*logit* as link). Next, using the coefficients computed for such models, the response for each patient of the remaining test set was predicted and compared with the real response in order to calculate the ROC curve. We iterated these operations for 10,000-times and computed the mean AUCs and the mean of the best sensitivities and specificities.

## Additional files

**Additional file 1.** Methods.

**Additional file 2: Table S1.** Separation criteria for responder and non-responder groups in the datasets.

**Additional file 3: Table S2.** Overview of responders and non-responders across datasets where both DNA and RNA data are available.

**Additional file 4: Table S3.** Per-patient summary of clinical classification where both DNA and RNA data are available.

**Additional file 5: Figure S1.** High level workflow for ICI biomarker validation and combination. The N biomarkers were individually tested for each dataset, this test serving as a primary validation of the proposed performance. Mean accuracy was computed for each classifier in all available datasets. Combinatorial analysis was carried out with majority voting and Generalized Linear Models. The E[X] formula stands as an example of the model to create when estimating the a0…aN linear factors.

**Additional file 6: Table S4.** Performance of each marker in the single datasets.

**Additional file 7: Table S5.** Summary of the biomarker performance. The second column indicates the number of datasets on which the marker was tested; the last column is the difference between the maximum and the minimum AUC for each biomarker.

**Additional file 8: Table S6.** Pearson correlation coefficients among each marker.

**Additional file 9: Table S7.** Combinations of uncorrelated markers and evaluation according to majority voting algorithm. For each combination, number of markers involved and AUC are shown.

**Additional file 10: Table S8.** Marker frequencies in the previous combinations with AUC greater than or equal to 0.65.

**Additional file 11: Table S9.** GLM coefficient estimates, with their respective standard errors, t-values and p-values.

## Abbreviations

CTLA4: cytotoxic T-lymphocyte associated protein 4
PD-1: programmed cell death protein 1
PD-L1: programmed death-ligand 1
PD-L2: programmed death-ligand 2
mAb: monoclonal antibody
ICI: immuno-checkpoint inhibitor
NSCLC: non-small-cell lung cancer
IHC: immunohistochemistry
IPS: immunophenoscore
IFN-γ: interferon gamma
ICB: immune checkpoint blockade
CTL: cytotoxic T lymphocytes
WES: whole exome sequencing
FPKM: fragments per kilobase million
TPM: transcripts per kilobase million
RECIST: response evaluation criteria in solid tumors
LB: long-term clinical benefit
NB: minimal or no benefit
CR: complete responder
PR: partial responder
SD: stable disease
PD: progressive disease
OS: overall survival
AUC: area under the curve
GLM: generalized linear model
ROC: receiver operating characteristics
