# Supplementary material for: Combinations of immuno-checkpoint inhibitors predictive biomarkers only marginally improve their individual accuracy
Source: J Transl Med. 2019 Apr 23;17:131. doi: 10.1186/s12967-019-1865-8 (PMC6480695; doi:10.1186/s12967-019-1865-8)
Supplement: Supplementary file 1 — Additional file 1. Methods. [file 12967_2019_1865_MOESM1_ESM.docx]

**Additional file 1: Methods**

**Genomic data harmonization**

We did not extract raw sequencing data from Exome and RNA-seq that would have allowed us to compute the neoantigen load [23], but we stuck to simpler measures from the primary analysis of genomic data. That is, mutation lists for Exome Sequencing and normalized expression at the gene level for RNA-seq (Fragments per Kilobase Million, FPKM, or Transcripts per kilobase Million, TPM), since our goal here was to establish whether there could be a way to develop a model classify for ICI response via simple quantification of high throughput DNA/RNA molecules from available patient's samples. Furthermore, even if the lack of raw data is limiting us from creating a bigger set of pooled patients (e.g. comprehensive melanomas) via sequence re-analysis, we think that this effort would have been vain in the light of the several dramatic confounding factors, the first one being the clinical separation of responders.

**Clinical Response in available datasets**

When comparing clinical response in our datasets, [19] and [8] used Response Evaluation Criteria in Solid Tumors (RECIST) 1.1 [23], while [7] used immune-related RECIST (irRECIST) 1 and 2 [24,25]; nothing is specified by [20] and [21]. Moreover, even if all the classifiers had the same output categorization (except for [21] in which there is only separation in long-term clinical benefit, LB, and minimal or no benefit, NB), there is no concordance about separation of patients who responded to immunotherapy (positive) or not. An overview of the variability among clinical classifications and our own separation method are provided in Table S7.

Progression free survival (PFS) and/or overall survival (OS) could have been included in the separation criteria but this information is not available in all the datasets. However, the real issue is how to define a PFS/OS threshold value using an independent method with respect to response. Going into detail of separation methods used in our datasets, [7] included complete responders (CR), partial responders (PR) and stable disease (SD) in the responder groups, while in the non-responder one, only progressive disease (PD) are present. [19] divided samples in three groups: PD, SD and CR/PR. [20] included in the positive group PR or SD lasting more than 6 months. We could not find or retrieve such information for [21]. Finally, for [8] in the responder group there were CR, PR and SD with OS greater than 1 year.

Table S8 contains the division among responders and non-responders according to our classification per dataset, while Table S9 describes the classification and the available OS data per patient. Both tables refer only to studies where both DNA and RNA data are available, since the combinatorial methods employed markers from both data types.
