# Supplementary figures and images for: Combinations of immuno-checkpoint inhibitors predictive biomarkers only marginally improve their individual accuracy
Source: J Transl Med. 2019 Apr 23;17:131. doi: 10.1186/s12967-019-1865-8 (PMC6480695; doi:10.1186/s12967-019-1865-8)

## Slide 1
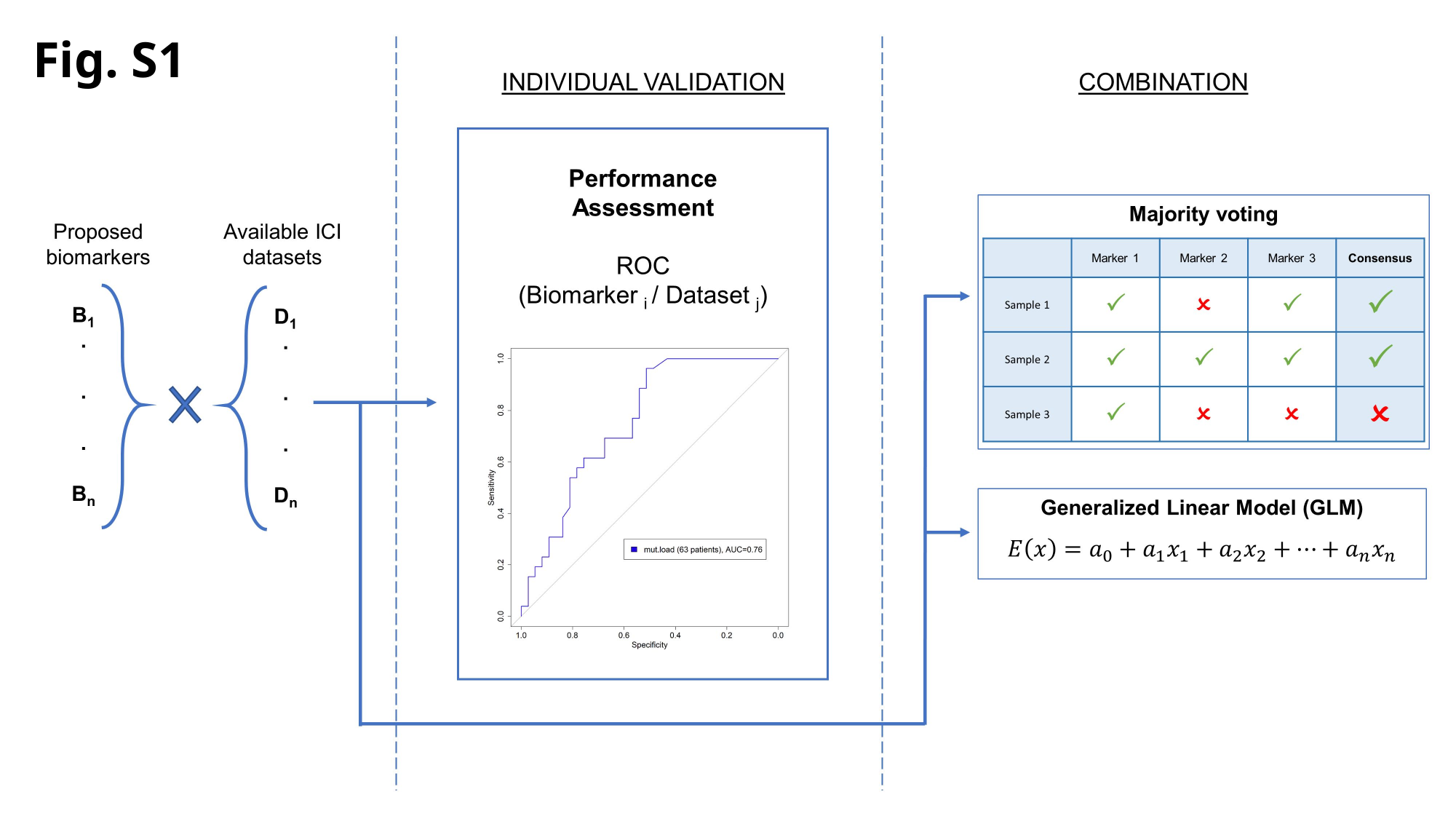

Fig. S1

Supplement: Supplementary file 5 — Additional file 5: Figure S1. High level workflow for ICI biomarker validation and combination. The N biomarkers were individually tested for each dataset, this test serving as a primary validation of the proposed performance. Mean accuracy was computed for each classifier in all available datasets. Combinatorial analysis was carried out with majority voting and Generalized Linear Models. The E[X] formula stands as an example of the model to create when estimating the a0…aN linear factors. [file 12967_2019_1865_MOESM5_ESM.pptx]
